# Survivability-Enhanced Virtual Network Embedding Strategy in Virtualized Wireless Sensor Networks

**DOI:** 10.3390/s21010218

**Published:** 2020-12-31

**Authors:** Dapeng Wu, Zhenli Liu, Zhigang Yang, Puning Zhang, Ruyan Wang, Xinqiang Ma

**Affiliations:** 1 School of Communication and Information Engineering, Chongqing University of Posts and Telecommunications, Chongqing 400065, China; s180101081@stu.cqupt.edu.cn (Z.L.); D180101013@stu.cqupt.edu.cn (Z.Y.); zhangpn@cqupt.edu.cn (P.Z.); wangry@cqupt.edu.cn (R.W.); 2 Key Laboratory of Optical Communication and Networks, Chongqing 400065, China; 3 Key Laboratory of Ubiquitous Sensing and Networking, Chongqing 400065, China; 4 School of Artificial Intelligence, Chongqing University of Arts and Sciences, Chongqing 402160, China; xinqma@cqwu.edu.cn

**Keywords:** wireless sensor network, network virtualization, survivable virtual network embedding, node reliability, resource reservation

## Abstract

With the widespread application of wireless sensor networks (WSNs), WSN virtualization technology has received extensive attention. A key challenge in WSN virtualization is the survivable virtual network embedding (SVNE) problem which efficiently maps a virtual network on a WSN accounting for possible substrate failures. Aiming at the lack of survivability research towards physical sensor node failure in the virtualized sensor network, the SVNE problem is mathematically modeled as a mixed integer programming problem considering resource constraints. A heuristic algorithm—node reliability-aware backup survivable embedding algorithm (NCS)—is further put forward to solve this problem. Firstly, a node reliability-aware embedding method is presented for initial embedding. The resource reliability of underlying physical sensor nodes is evaluated and the nodes with higher reliability are selected as mapping nodes. Secondly, a fault recovery mechanism based on resource reservation is proposed. The critical virtual sensor nodes are recognized and their embedded physical sensor nodes are further backed up. When the virtual sensor network (VSN) fails caused by the failure physical node, the operation of the VSN is restored by backup switching. Finally, the experimental results show that the strategy put forward in this paper can effectively guarantee the survivability of the VSN, reduce the failure penalty caused by the physical sensor nodes failure, and improve the long-term operating income of infrastructure provider.

## 1. Introduction

Nowadays, Wireless Sensor Network (WSN) has been widely used in monitoring, controlling, tracking and other fields based on its sensing, computing and communication capabilities [1]. In order to help realize the utilization potentiality of the Internet of Things (IoT), the WSN virtualization architecture is emerging to overcome the inefficiencies of proprietary, single-purpose, single-user WSNs [2,3]. Driven by the developing needs of the IoT, the 5G mMTC scenario should deploy a huge number of sensors [4,5,6], while the traditional WSN in the public area is generally laid separately by each user for his specific task, which is unavailable for other users even if the state of WSN is idle. Some other sensing nodes need to be deployed when the user performs other types of sensing tasks, which leads to the high cost and low reuse rate. By virtualizing the physical node resources, such as sensing resources and processing capabilities, and link resources of WSN like wireless channel resources, the existing WSN can be regarded as a shared multi-user perception infrastructure, enabling them to configure multiple coexistence virtual sensor network (VSN) on demand [7].

Through node-level and network-level virtualization, the virtualized WSN decouples the service role of traditional WSN into a wireless sensor network infrastructure provider (WSNInP) and virtual sensor network service provider (VSNSP), where WSNInP is responsible for deploying, managing and maintaining WSN [8]. VSNSP leases sensing resources from WSNInP, assigns sensing tasks to physical nodes and provides users with on-demand services. VSNSP coordinates and collaborates with multiple VSNs in an overlapping way. Each VSN represents an application with a different performance level defined by users in the application layer, which effectively alleviates the problems of low resource utilization and poor task execution flexibility in the current WSN [9,10].

One of the key technologies of WSN virtualization is virtual network embedding (VNE), which effectively maps VSN to the underlying physical network. The essence of VNE is the process of allocating a physical WSN network resources to VSN. Through virtualization technology, VSNs achieves effective and reasonable sharing of underlying physical network resources, which increases the revenue of WSNInP. The optimal VNE problem has been proven to be NP-hard [11] issue. Many researchers have proposed several heuristic algorithms. Most of them focus on the request acceptance rate, the underlying resource utilization and theaverage revenue, without considering the survivability of the embedded virtual network [12]. In actual situations, physical sensor nodes may malfunction or deteriorate due to human or non-human factors. Due to the characteristics of resource sharing in network virtualization, multiple VSNs share the same underlying physical infrastructure, thus the breakdown of one physical node will make the VSNs whose virtual nodes mapped to it invalid, resulting in affecting related sensing services. WSNInP must bear the compensation specified in the service level agreement (SLA) due to the failure of the VSN, which will cause huge economic and reputation losses to the WSNInP. Hence, it is a vital issue that how to efficiently and reasonably embed the VSN and provide a robust and uninterrupted network service.

To guarantee the survivability of VSN, sufficient physical nodes and link resources should be reserved for each VSN during instantiation. Once a physical node fails, the failed virtual node and link can be quickly remapped to the standby physical resources to ensure the normal operation of the VSN. However, the reservation for each VSN will greatly reduce the utilization rate of WSNInP physical infrastructure. Considering the limited sensing range and resources of sensor nodes, the resource sharing method in cellular networks is unsuitable for virtualized sensor networks [13]. Thus, how to perform backup resource allocation for VSN to balance the effectiveness and the reliability of VSN when performing survivable embedding is crucial.

The existing work about WSN virtualization hardly considers the survivability guarantee of a virtualized sensor network. The problem of multiple virtual network failures caused by a single physical node failure has not been effectively resolved. Therefore, based on the above research deficiencies, this paper puts forwards a survivability-enhanced virtual network embedding strategy in a virtualized wireless sensor network. Compared with the existing work [12], which only considers a single link failure, this paper further considers the failure of multiple VSNs caused by a single physical node failure, and effectively solves this problem. Aiming at the failure of a single physical node, the SVNE problem is modeled as a mixed-integer programming problem based on the characteristics of WSN virtualization in this paper. A heuristic algorithm NCS is proposed. The main contributions of this paper are summarized as follows:

(1) A survivable virtual network embedding model is established. Considering the resource consumption of nodes and links during the initial mapping and failover, the problem is transformed into the problem of minimizing link consumption to maximize the WSNInP’s long-term revenue.
(2) A novel node reliability-aware virtual sensor network embedding method is proposed. The resource reliability of physical sensor nodes is evaluated according to node failure rate and congestion degree. Then the one with higher reliability is selected to embed a virtual node to improve the acceptance rate of VSNR and reduce the failure probability of physical nodes that perform VSN tasks.
(3) A failure recovery strategy combining the protection mechanism and recovery mechanism is proposed to improve the VSN failure recovery rate. Resource backup is performed for the critical virtual sensor nodes. When a failure occurs, the affected VSN will be restored with the least spare resources to guarantee the survivability of the VSN and increase the long-term operating profit of the WSNInP.

The remainder of this paper is organized as follows. The related research works are introduced in Section 2. Section 3 presents the system model and SVNE problem statement. The embedding strategy NCS is described in Section 4. Then, the experimental results and analysis are given in Section 5. Finally, Section 6 concludes the paper and discusses future work.

## 2. Related Work

### 2.1. WSN Virtualization

In recent years, with the developments of the IoT, virtualization in WSN has received widespread attention. Some potential measures have been taken for improving the flexibility and scalability of WSN deployment and the investment return rate of WSNInP. Literature [9] divided virtualization technologies into two categories, node-level and network-level. Node-level virtualization is to abstract a single sensor node to overcome the application’s platform dependency and code modularization problems. In this field, virtual machine-based architectures have been proposed to achieve virtualization and reprogrammability, such as MATE, ASVM, Melete and VMStar, which are frameworks for building application-specific virtual machines on constrained sensor platforms. Network-level virtualization usually includes two main building blocks, a management platform that supports multiple applications sharing the physical WSN infrastructure, and tools or algorithms that allocate physical resources for multiple applications. For instance, the management platforms, SenHare and UMADE, have created multiple overlay sensor networks and allow different applications to share the same physical infrastructure.

Based on the above research, literature [14] presented a software-defined sensor network virtualization architecture, which enhanced the flexibility of network re-orchestration via virtualizing. Its framework is based on Industry 4.0 for exploring the ability of the WSN network virtual environment to re-coordinate the node functions and the entire network operation level. Literature [1] devised an architecture to reduce redundant deployment of sensor networks for diverse IoT applications. The architecture contained four layers, physical layer, virtual sensor layer, virtual sensor access layer, and application layer. It also introduced a new software framework for WSN virtualization and defined the division of labor between various stakeholders after the process of WSN virtualization.

Based on various virtualized WSN architectures, most of the related work focuses on resource optimization, which is to abstract physical sensors based on application requirements, and improve resource utilization by executing multiple application-centered tasks in sensors. For example, literature [15] considered both the sensor energy consumption and resource utilization, which allowed the same physical sensor to perform multiple tasks in the WSN virtualization environment. Literature [16] modeled and analyzed the joint problem of multi-task control admission and physical resource allocation in virtual WSN. A software-defined WSN prototype was proposed in [17] to centrally control the routing in the dynamic network to improve energy efficiency.

### 2.2. Virtual Network Survivable Embedding

Currently, there are two main survivability guarantee mechanisms, protection mechanism and recovery mechanism. The protection mechanism further contains two kinds of methods, the active protection method and the passive protection method. The active one means pre-allocating spare underlying network resources for the virtual network. When mapping the virtual network, the working resources and the spare resources are simultaneously mapped. Literature [18] constructed an auxiliary protection graph to improve the survivability of a single node according to the initial virtual network request. The passive protection denotes that when the underlying network node or link fails, the backup resource reserved in advance is used to restore the virtual network service rather than mapping work resources and backup resources at the same time. Literature [19] solved the link failure problem by constructing a set of backup paths for each link. Literature [20] jointly optimized the spare capacity allocation and virtual network embedding in the virtual network, so that the bandwidth capacity was guaranteed when there were multiple substrate link failures. However, when the network normally runs, it is undoubtedly a huge waste to provide redundant resources backup for the virtual link, and the utilization of the underlying physical resources will be greatly reduced. At the same time, the success rate of subsequent VSN mapping and the long-term benefit of WSNInP will be affected.

The recovery mechanism does not provide backup resources for the virtual network. After a node or link fails, the virtual nodes and links affected by the failure are migrated, and the node and link resource are redistributed to restore virtual network services. Literature [21] proposed a remapping method based on coordination game theory and described the initial virtual network mapping and subsequent fault recovery remapping as two staggered coordination games, so as to achieve the optimal Nash equilibrium between infrastructure providers and network service providers during fault recovery. Literature [22] identified the main cut sets from the sub-networks of the virtual network after removing the faulty node and then adopted the ant colony algorithm to select nodes and links to search for the best embedding of the virtual network. However, the recovery mechanism shows a lower recovery success rate when the underlying free resources are less.

### 2.3. Survivable Embedding in a Virtulized Sensor Network

A key way to solve the WSN fault problem is to use a redundancy mechanism to eliminate the impact of the fault. Literature [23] proposed the concept of fault tolerance, which guaranteed the fault tolerance capability of the WSN by ensuring that there were at least k disjoint paths between any two nodes in the network. Literature [24] realized the fault tolerance towards WSN via using node redundancy, which denoted that before node failure occurs, redundant nodes could be found in the network topology through adopting the attribute matching principle. After the node failed, the redundant node replaced the failed node and took over the sensing task. However, the research on the survivability of VSN towards the WSN virtualization is scarce. Literature [12] presented a fault-tolerance framework oriented to heterogeneous networks and adopted a genetic algorithm to carry out post-fault recovery for WSN links, modeling fault tolerance and communication delay as two conflicting objectives in an optimization problem. However, it performs a low success rate of link recovery when the underlying resource is saturated, and cannot recover the link failure caused by the source sensor node failure.

Therefore, this paper proposes a novel VSN survivability embedding architecture and heuristic algorithm. The node reliability is firstly assessed and then suitable nodes with higher reliability are chosen for reliable mapping. Furthermore, according to the degree of nodes in the VSN, the criticality for each virtual sensor node is evaluated, and resource reserved is performed for critical virtual sensor node. When a physical sensor node fails, the critical virtual sensor node is switched to the backup node, or find a feasible migration node in the remaining network for the normal virtual sensor node.

## 3. System Model and Problem Formulation

### 3.1. Network Model

**Figure 1:**
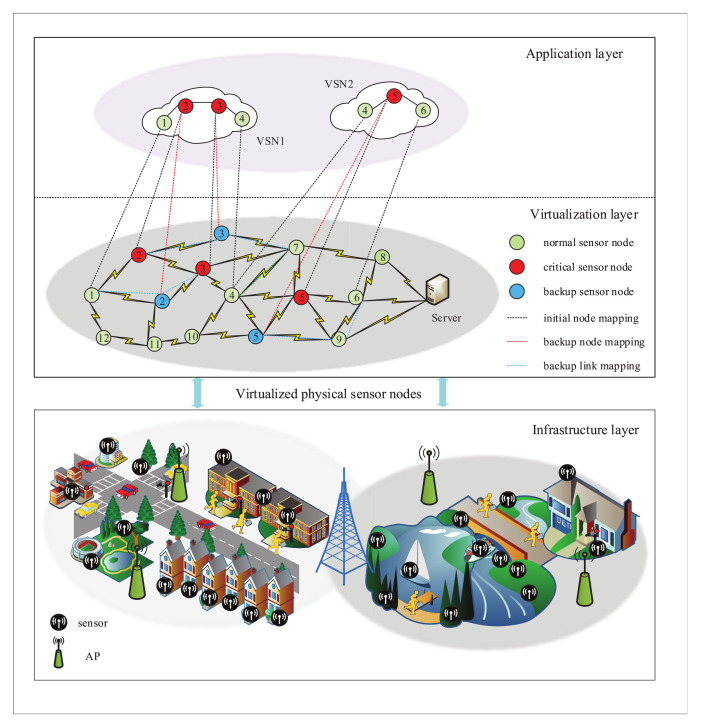
Virtualized wireless sensor network (WSN) survivable embedding model diagram.

As shown in Figure 1, the virtualized WSN consists of three layers, the infrastructure layer, the virtualization layer and the application layer. In the infrastructure layer, the physical network of WSN includes a sensor control server and a group of heterogeneous sensor nodes regulated by WSNInP. Each node is integrated with multiple types of sensors, such as temperature sensors, humidity sensors, and infrared sensors, etc. [25]. Each sensor is responsible for the specific sensing task of the corresponding target group in its sensing area. To facilitate the theoretical analysis, the physical network of WSN is represented by an undirected graph $G^{S}=\left(N^{S},L^{S}\right)$, where $N^{S}$ and $L^{S}$ represent the set of physical nodes and the set of links between physical nodes in the WSN, respectively. The traffic volume in the WSN is first transmitted to the APs served as sink nodes and then to the controller, which controls and manages the real-time task resource allocation. For each physical node $n_{i}^{s}\in N^{S}$, the attribute category $R^{s}=\left\{c\left(n_{i}^{s}\right),m\left(n_{i}^{s}\right),loc\left(n_{i}^{s}\right),E\left(n_{i}^{s}\right)\right\}$ denotes the global network resources, where $c(n_{i}^{s})$, $m(n_{i}^{s})$, $loc(n_{i}^{s})$, and $E(n_{i}^{s})$ respectively mean the computing capacity, the storage capacity, the geographic location, and the energy of the physical sensor node. Meanwhile, for any link $l_{ij}^{s}\in L^{S}$, the link capacity between a pair of nodes $\left(n_{i}^{s},n_{j}^{s}\right)$ is $b(l_{ij}^{s})$.

Similarly, the VSN is also described by an undirected graph $G^{V}=\left(N^{V},L^{V}\right)$, where $N^{V}$ and $L^{V}$ represent the set of virtual sensor nodes and virtual links in the VSN, respectively. $\omega =\{\omega_{1},\omega_{2},\cdots ,\omega_{n}\}$ denotes a group of real-time tasks accommodated over VSNs, in which each request corresponds to different services with differentiated resource requirements. The attribute item $R^{V}=\left\{c\left(n_{i}^{v}\right),m\left(n_{i}^{v}\right),loc\left(n_{i}^{v}\right)\right\}$ defines the resources required by the virtual sensor request, where $c(n_{i}^{v})$, $c(n_{i}^{v})$ and $loc(n_{i}^{v})$ demonstrates the computing capacity, the storage capacity, and the sensing position required by the virtual node, respectively. For any virtual link $l^{v}\in L^{V}$, the link capacity between a pair of virtual nodes $\left(n_{i}^{v},n_{j}^{v}\right)$ is $b(l_{ij}^{v})$.

### 3.2. Physical Network Failure

In general, it is assumed that VSN embedding occurs under the normal operation of the underlying physical sensor network [9]. However, it is inevitable that the underlying physical sensor network will occasionally fail due to the situation of running out of energy, wireless channel interruption, hardware damage, embedded operating system or application software crash, and so on. Compared with the link failure, it is more complicated to ensure the survivability of the VSN in the case of node failure. Leveraging on such premises, safeguarding the survivability of the VSN requires not only the backup node but also the allocation of additional resources for the required links. Therefore, this paper considers VSN survivability embedding for physical sensor node faults.

When the physical sensor node $n_{i}^{s}$ fails, both the virtual sensor node hosted on the node and the virtual link containing $n_{i}^{s}$ will fail. The former is called virtual node failure and the latter is called virtual link failure. The processing method of virtual link failure is similar to that of a physical link failure, which can be solved by utilizing SVNE methods [12]. Thus, we focus on studying the survivable embedding method for the first failure scenario.

From the perspective of the time domain, the WSN physical node faults can be divided into transient faults and permanent faults. Transient faults refer to a type of faults that will automatically recover and have a short duration after the fault occurs, such as accidental reset of nodes caused by electromagnetic interference. While permanent faults are defined as a type of failure that is irrecoverable after the fault occurs, such as running out of energy, hardware damage, etc. We assume that node failure is a single failure model, which means that only one failure occurs in the physical WSN network at the same time. The physical network node failure is described from two dimensions, the failure happen time and the mean recovery time. The failure happen time follows Poisson distribution and the mean recovery time follows a geometric distribution. The physical sensor node failure is modeled as a series of single node failures, denoting $F_{1},\cdots ,F_{n}$ in chronological order. For node failure $F_{i}$, $s(F_{i})$ and $e(F_{i})$ represent its occurrence time and end time, respectively. When a node fails instantaneously, $s\left(F_{i}\right)<e\left(F_{i}\right)$, which means $F_{i}$ will last for a certain time. When a node fails permanently, $e(F_{i})=\infty$.

The distributed fault detection mechanism is currently the mainstream direction of WSN fault detection research [17]. Fault detection of sensor nodes is performed locally by each sensor node instead of being handled centrally by the controller. When the sensor node fault is detected, new tasks will no longer be scheduled to the known failed sensor node.

### 3.3. SVNE Problem Formulation

It is a vital step of WSN virtualization to efficiently embed VSNs onto the substrate WSN. The mapping process is divided into two stages, node mapping and link mapping. In the first stage, node mapping is implemented to find appropriate physical sensor nodes for the sensing task requested by VSNR to embed virtual sensor nodes. In the second stage, link mapping is carried out on the basis of the first stage. Feasible communication paths are constructed for the physical nodes that have already been embedded onto the virtual node. In addition, it is also important to ensure that the resource constraints of the VSNR on the physical sensor node and link request must be met.

However, if the VSN mapping is only considered from the perspective of computing and sensing resource constraints, once a physical sensor node with a higher failure rate is selected, the survivability of all VSNs mapped on it will be affected. Hence, when selecting physical sensor nodes, under the resource constraints of VSNR, nodes with high reliability should be given high mapping priority, which reduces the probability of service interruption caused by physical sensor node failure in VSN. In addition, in order to guarantee the survivability of the VSN, a backup node is set up to deal with the failure of the underlying physical sensor node that occurs during the mission. When the physical sensor node fails, the VSNSP can quickly switch to the backup node to maintain the normal operation of the VSN. Therefore, the SVNE problem can be modeled as follows:

The binary variable $r_{ij}\in \left\{0,1\right\}$ denotes the mapping relationship between the virtual sensor node $n_{i}^{v}$ and the physical sensor node $n_{j}^{s}$. If the virtual sensor node $n_{i}^{v}$ is mapped to the physical sensor node $n_{j}^{s}$, $r_{ij}=1$. Otherwise $r_{ij}=0$. Similarly, the binary variable $f_{ij\to mn}\in \left\{0,1\right\}$ represents the mapping relationship between the virtual link $l_{ij}^{v}$ and the physical link $l_{mn}^{s}$. When the virtual network is mapped, the storage and computing resources of the virtual sensor nodes are fixed, while the resource consumption of virtual links may vary greatly due to the prominent differences in physical paths. Therefore, one challenging issue for performing VSN mapping and remapping lies in how to minimize link resource consumption.

(1) $$min\sum_{l_{mn}^{s}\in L^{S}}\sum_{l_{ij}^{v}\in L^{V}}f_{ij\to mn}\times \left(b\left(l_{ij}^{v}\right)\right)+\sum_{l_{ab}^{s}\in L^{S}}\sum_{l_{ij}^{v}\in L^{V}}f_{ij\to ab}\times \left(b\left(l_{ij}^{v}\right)\right)$$

(2) $$\sum_{n_{i}^{v}\in N^{V}}r_{ij}=1,\forall n_{j}^{s}\in N^{S}$$

(3) $$\sum_{n_{j}^{s}\in N^{S}}r_{ij}=1,\forall n_{i}^{v}\in N^{V}$$

(4) $$r_{ij}\times c\left(n_{i}^{v}\right)\leq Rc\left(n_{j}^{s}\right),\forall n_{i}^{v}\in N^{V},\forall n_{j}^{s}\in N^{S}$$

(5) $$r_{ij}\times m\left(n_{i}^{v}\right)\leq Rm\left(n_{j}^{s}\right),\forall n_{i}^{v}\in N^{V},\forall n_{j}^{s}\in N^{S}$$

(6) $$f_{ij\to mn}\times b\left(l_{ij}^{v}\right)\leq Rb\left(l_{uv}^{s}\right),\forall l_{ij}^{v}\in L^{V},l_{uv}^{s}\in L^{S}$$

(7) $$\sum_{n_{m}^{s}\in A\left(x\right)}(f_{ij\to xm}+f_{ij\to mx})=0,\forall i,j\in N^{V}$$

(8) $$f_{ij\to yn}\left(Rb\left(l_{yn}^{s}\right)-Rb^{\prime }\left(l_{yn}^{s}\right)\right)=r_{iy}b\left(l_{ij}^{v}\right),\forall i,j\in N^{V}$$

In Equation (1) the $\sum_{l_{mn}^{s}\in L^{S}}\sum_{l_{ij}^{v}\in L^{V}}f_{ij\to mn}\times \left(b\left(l_{ij}^{v}\right)\right)$ is the resource consumption of the initial reliable mapping, and the $\sum_{l_{ab}^{s}\in L^{S}}\sum_{l_{ij}^{v}\in L^{V}}f_{ij\to ab}\times \left(b\left(l_{ij}^{v}\right)\right)$ is the resource consumption of the link remapping during failure recovery. Equations (2)–(8) are embedding constraints. Equations (2) and (3) are the independence constraints demonstrating that each virtual sensor node of VSNR is mapped into an independent physical sensor node in a one-to-one manner. Equation (2) ensures that the same physical node can only accommodate one virtual sensor node in the same VSNR, and Equation (3) ensures that a virtual sensor node is mapped onto only one physical sensor node. Equation (4) is the node computing capacity constraint of VSN mapping. When the virtual sensor node is initially mapped or remapped, the computing resource demand of the virtual sensor node cannot be greater than $Rc(n_{j}^{s})$, which denotes the remaining computing resource of the physical sensor node. Similarly, Equations (5) and (6) are the storage capacity constraint and link transmission capacity constraint of the VSN, respectively. Equation (7) indicates that the physical link connected to the faulty physical sensor node $n_{x}^{s}$ does not participate in the remapping process. A(x) represents the set of adjacent nodes with the fault physical sensor node $n_{x}^{s}$. Equation (8) indicates that if $n_{i}^{v}$ is remapped to $n_{y}^{s}$, the output traffic of the node $n_{y}^{s}$ is equal to the data transmission request of the link $l_{ij}^{v}$, which is the starting point of the physical link hosting the virtual link $l_{ij}^{v}$. $Rb(l_{yn}^{s})$ and $Rb^{\prime }\left(l_{yn}^{s}\right)$ are the remaining link resources of the node $n_{y}^{s}$ before and after remapping, respectively.

### 3.4. Performance Metrics

This paper proposes an online VSN embedding algorithm with the constraints of the physical WSN to maximize the long-term average revenue of WSNInP while guaranteeing high-quality services and providing users with stable services. Therefore, this paper introduces the request acceptance rate, failure recovery rate and WSNInP long-term revenue of VSNR embedding as the evaluation index of VSNR embedding.

#### A. Virtual sensor network request acceptance rate

VSNR acceptance rate is defined as the ratio of the number of VSNR accepted to the total number of VSNR arrived during the time τ.

(9) $$\eta =\lim_{\tau \to \infty }\;\frac{n_{1}\left(\tau \right)}{n_{2}\left(\tau \right)},$$

where $n_{1}\left(\tau \right)$ denotes the number of VSNRs accepted during the time τ, and $n_{1}\left(\tau \right)$ is the number of VSNRs arrived during the time τ.

#### B. Recovery rate of virtual sensor network

The VSN recovery rate refers to the ratio of the number of VSNs recovered successfully to the total number of VSNs caused by fail physical sensor nodes.

(10) $$\xi =\lim_{\tau \to \infty }\;\frac{F_{1}\left(\tau \right)}{F_{2}\left(\tau \right)},$$

where $F_{1}\left(\tau \right)$ is the number of VSN recovered successfully during the time τ, and $F_{1}\left(\tau \right)$ is the total number of invalid VSN caused by physical sensor node failure during the time τ.

#### C. Long-term benefits of WSNInP

In order to increase long-term operating profit, WSNInP needs to consider two key factors when providing services: the revenue obtained by mapping the VSN and the compensation that must be paid when the VSN becomes invalid due to a failure according to the SLA.

In general, the benefits of WSNInP accepting VSNRs depend on the duration of the VSN and the required underlying network resources. $T(G^{V})$ describes the active time that VSN needs to work continuously, $p_{price}$ defines the unit price of the resource, $B(l_{ij}^{v})$ denotes the bandwidth requirement of the virtual link, $C(n_{i}^{v})$ represents the computing capacity requirement of the virtual sensor node. Therefore, the revenue $R(G^{V})$ of VSNSP accepting VSNRs is defined as follows.

(11) $$R\left(G^{V}\right)=T\left(G^{V}\right)p_{price}\left(\sum_{n_{i}^{v}\in N^{V}}C\left(n_{i}^{v}\right)+\sum_{l_{ij}^{v}\in L^{V}}B(l_{ij}^{v})\right).$$

WSNInP needs to consume underlying network resources to accept VSNRs. To ensure the reliability of the mapping the backup resources will consume additional resources. $p_{cost}$ is the unit cost of the resource, $B(l_{mn}^{s})$ is the link resource of the physical link corresponding to a virtual link, $C_{pri}\left(n_{i}^{s}\right)$ is the leased resource required by the initial intact VSN to provide services, and $C_{backup}\left(n_{i}^{s}\right)$ is the CPU resource of the backup node reserved for reliability requirements. Therefore, the cost of WSNInP when accepting VSN is expressed as follows.

(12) $$C\left(G^{V}\right)=T\left(G^{V}\right)p_{cost}\left(\sum_{n_{i}^{s}\in N^{S}}C_{pri}\left(n_{i}^{s}\right)+\sum_{n_{i}^{s}\in N^{S}}C_{back}\left(n_{i}^{s}\right)+\sum_{i=1}^{H}\sum_{l_{ij}^{s}\in L^{S}}B(l_{ij}^{s})\right).$$

When a physical sensor node fails, WSNInP considers virtual node migration by selecting backup node resources to ensure the survivability of VSN. As shown in Figure 1, when the physical sensor node fails, the backup node can be selected in the virtual backup resource pool to remap and continue to provide services for users. At this time, WSNInP only needs to consume additional backup resources instead of paying the penalty. If there is no suitable candidate node resource or the migration of the failed node resource fails, the VSN becomes invalid. WSNInP needs to bear the compensation $p(G^{V})$ specified for the invalid $VSN_{i}$ in the SLA. The compensation $P(n_{i}^{s})$ that WSNInP must pay, due to the failure of the physical node $n_{i}^{s}$, is given as follows.

(13) $$P\left(n_{i}^{s}\right)=\sum_{G^{V}\in D\left(n_{i}^{s}\right)}p(G^{V}),$$

where $D(n_{i}^{s})$ is the set of invalid VSNs due to the fault physical sensor node $n_{i}^{s}$.

Let Revenue(τ) denote the long-term operating income of WSNInP during the time τ, which refers to the income obtained by providing services minus the compensation caused by the invalid VSN during this period.

(14) $$Revenue\left(\tau \right)=\sum_{G^{V}\in M\left(\tau \right)}(R\left(G^{V}\right)-C\left(G^{V}\right))-\sum_{n_{i}\in B\left(\tau \right)}^{H}P(n_{i}^{s}),$$

where M(τ) defines the set of VSN successfully mapped during the time τ, and B(τ) represents the set of physical sensor nodes that failed during the time τ.

## 4. NCS Survivable Embedding Algorithm

In order to enhance the survivability of VSN, this section comprehensively considers multiple factors that affect the reliability of physical sensor nodes and proposes NCS. It guarantees the survivability of VSN from two perspectives. First, for node selection, the physical sensor nodes with higher reliability are screened out to carry virtual sensor nodes through node reliability perception. Second, from the perspective of failure recovery, when the initial mapping is completed, a backup node is constructed for the physical node mapped by the critical virtual sensor node of the VSN to deal with the problem of node failure.

### 4.1. Node Reliability-Aware Embedding

In the virtual node mapping stage, in addition to considering whether physical sensor node resources can meet the requirements of virtual sensor nodes, physical node reliability should also be considered. The virtual sensor nodes are mapped onto relatively more reliable physical nodes to further reduce the failure probability of VSN.

Node reliability can be perceived in terms of failure rate and congestion degree. In terms of failure rate, reliability cost is an important indicator. Reliability is defined as the probability that a real-time task will still work even if there is a hardware failure [26]. Assuming that the failure arrival rate is constant, and the Poisson distribution is used to estimate the distribution of failure counts in any fixed time interval. The reliability cost can be defined as Equation (15).

(15) $$rc=\sum_{j=1}^{m}\sum_{i=1}^{n}\lambda_{j}et_{ij},$$

where $et_{ij}$ is the execution time of the task $t_{i}$ on the sensor node $n_{{}_{j}}^{s}$ and $\lambda_{j}^{s}$ is the failure rate of the node $n_{{}_{j}}^{s}$.

The reliability of the WSN network for a set of real-time tasks can be expressed as Equation (16).

(16) $$tr=e^{-rc}.$$

However, based on the assumption that components have a constant failure rate, it usually leads to an inaccurate estimate of the failure probability of physical nodes, which means that their performances do not degrade over time. Therefore, this paper considers the failure frequency of the node. The more historical failures the physical sensor node has, the more unstable the performance of the node is. Importantly, the reliability of the WSN physical sensor node is updated to Equation (17).

(17) $$tr=e^{-rc}/\left(fn\left(n_{i}^{s}\right)+1\right),$$

where $fn(n_{i}^{s})$ represents the number of historical failures of the physical sensor node $n_{i}^{s}$.

From the perspective of congestion, if the data volume generated and received by the node exceeds the upper limit of its forwarding data capacity, it will cause node congestion, network load imbalance, and greatly increase service delay and packet loss rate [7]. Mapping virtual sensor nodes onto physical sensor nodes with more remaining available resources is conducive to improve the load balancing of the network and to enhance the reliability of VSN to a certain extent. The available resource ratio is defined as the ratio of the available resources of the node to the total resources of the node, as shown in Equation (18).

(18) $$RS\left(n_{i}^{s}\right)=\frac{Rc\left(n_{i}^{s}\right)Rb\left(n_{i}^{s}\right)}{c\left(n_{i}^{s}\right)b\left(n_{i}^{s}\right)}.$$

During node mapping, selecting nodes with high reliability can avoid mapping nodes onto physical sensor nodes with more failures, which will reduce the failure probability of mapped physical sensor nodes, and guarantee the survivability of VSN. Considering the above factors comprehensively, the node reliability is defined as Equation (19).

(19) $$RD\left(n_{i}^{s}\right)=tr\cdot RS\left(n_{i}^{s}\right).$$

The survivable node mapping algorithm is shown in Algorithm 1. When mapping VSNRs, we can sort the reliability of the underlying node resources in descending order. Priority is given to selecting physical nodes with higher reliability meeting the node constraints for critical virtual nodes. Then link mapping is performed to find a suitable path for the communication of the embedded VSN nodes, which is described in Algorithm 2. NCS takes advantage of the k-shortest path algorithm to perform virtual link mapping. Among the first k shortest paths, the virtual link with high data transmission capacity requirements in VSNR is first embedded in the physical link with the most remaining available resources.
**Algorithm 1** Node reliability-aware backup survivable embedding algorithm (NCS).**Input:** physical WSN $G^{S}$; VSNR $G_{T}^{V}$**Output:** The node mapping results set $NODE=\{n_{j}^{s},\cdots ,n_{k}^{s}\}$**Initialization:** Candidate node set Ns=∅, node mapping results set NODE=∅**for**$n_{i}^{v}$ in VSNR **do**       **if** $\sqrt{loc\left(n_{i}^{v}\right)-loc\left(n_{j}^{s}\right)}\leq M$ **then**
         Add $n_{j}^{s}$ to Ns**      end if****      if**Ns=∅**then**         
NODE=∅
**         return** Node embedding failed**      else**          **for** each $n_{j}^{s}$ in Ns **do**             **if** $c\left(n_{i}^{v}\right)\leq Rc\left(n_{j}^{s}\right)$ and $m\left(n_{i}^{v}\right)\leq Rm\left(n_{j}^{s}\right)$ **then**               Calculate $RD(n_{j}^{s})$ through Equation (19)         
**end if**
      
**end for**
      Choose $n_{j}^{s}$ with the highest $RD(n_{j}^{s})$      Add $n_{j}^{s}$ to NODE**   end if****end for****return** Node embedding successfully**Algorithm 2** NCS link embedding algorithm.**Input:** physical WSN $G^{S}$, VSNR $G_{T}^{V}$, node mapping results set $NODE=\{n_{j}^{s},\cdots ,n_{k}^{s}\}$**Output:** The link embedding results $LINK=\{l_{ab}^{s},\cdots ,l_{mn}^{s}\}$**Initialization:** The link embedding results LINK=∅**for**$l^{v}\in L^{V}$ in VSNR **do**      Choose $l_{ij}^{v}$ with the highest $b(l_{ij}^{v})$       **for** $l^{s}\in L^{S}$ **do**
          **if** $b\left(l_{mn}^{s}\right)<b\left(l_{ij}^{v}\right)$ **then**
            $L^{S}\leftarrow L^{S}/\left\{l_{mn}^{s},l_{nm}^{s}\right\}$
          **end if**
       **end for**
      K-shortest path algorithm for $n_{i}^{s}$ and $n_{j}^{s}$      Choose $l_{ab}^{s}$ with the highest $b(l_{ab}^{s})$      Add $l_{ab}^{s}$ to LINK**end for****return** link embedding successfully

### 4.2. VSN Failure Recovery Based on Resource Reservation

**Definition** **1.** *In a VSN, if the connectivity of the virtual sensor node $n_{i}^{v}$ is m times as much as the average network node degree, the node is defined as the critical virtual sensor node, and m represents the critical node determination coefficient.*

(20) $$deg\left(n_{i}^{v}\right)=\frac{m}{n}\sum_{j=1}^{n}deg(n_{j}^{v}),\;m\geq 1,\forall n_{i}^{v}\in CVN,$$

where CVN denotes the set of critical virtual sensor nodes, and $deg(n_{i}^{v})$ describes the degree of the critical virtual sensor node $n_{i}^{v}$.

When selecting appropriate backup physical sensor nodes for critical virtual sensor nodes, in addition to meeting the basic node mapping constraints mentioned in Section 3.3, the following three aspects should be also considered.

(1) Monitoring range constraints. The monitoring range of wireless sensor nodes is limited. In order to meet the sensing location request of the virtual sensor node, the monitoring range of the backup physical sensor node should cover the location request.

(21) $$\left|loc\left(n_{i}^{s}\right)-loc\left(n_{j}^{v}\right)\right|\leq R\left(n_{i}^{s}\right),$$

where $R(n_{i}^{s})$ is the sensing radius of the physical sensor node $n_{i}^{s}$.

(2) Connectivity. When a physical sensor node fails, the VSN facilities need to be remapped to resume normal operation. These virtual sensor facilities include: (a) the virtual sensor node carried on the physical sensor node; (b) the virtual link containing the physical node on the mapped physical path. Therefore, in the WSN network, if a physical sensor node $n_{i}^{s}$ is to become a backup node of another physical sensor node $n_{x}^{s}$, $n_{i}^{s}$ must be able to reach the other physical nodes embedded by the neighbor virtual sensor nodes of the critical virtual sensor node carried by $n_{x}^{s}$ within a certain geographic range. Since each hop in the routing will produce corresponding resource overhead and transmission delay, this paper adopts the number of hops to measure the geographic range and defines the set as (22).

(22) $$H\left(G^{S},n_{j}^{s},h\right)=\{n_{i}^{s}|\min\left(loc\left(n_{i}^{s}\right),loc\left(n_{j}^{s}\right)\right)=h,n_{i}^{s}\in G^{S}\setminus n_{x}^{s},n_{j}^{s}\in E\left(n_{k}^{v}\right)\},$$

where $G^{S}\setminus n_{x}^{s}$ represents the remaining physical network after removing the faulty node in the WSN network. $E(n_{k}^{v})$ denotes the physical node mapped by the neighbor virtual node of the critical virtual sensor node. Thus $H(G^{S},n_{j}^{s},h)$ is the candidate backup physical sensor set composed of the nodes who can reach the physical sensor node $n_{j}^{s}$ in *h* hops.

(3) Recovery capability. The recovery capability of the backup node is measured from the node similarity and the proportion of remaining available resources. As shown in formula (23), the node similarity of the node $n_{i}^{s}$ to node $n_{x}^{s}$ is the ratio of the number of their common neighbor nodes to the number of all neighbor nodes of $n_{x}^{s}$. The more common neighbors of $n_{x}^{s}$ and $n_{i}^{s}$ have, the more nodes connected to $n_{x}^{s}$ can be covered by $n_{i}^{s}$ as a backup node, which can better maintain the fault tolerance of the WSN network to avoid VSN remapping failure.

(23) $$NS\left(n_{i}^{s}\right)=\lambda_{i}^{s}\frac{\left|H\left(G^{S},n_{i}^{s},1\right)\cap H\left(G^{S},n_{x}^{s},1\right)\right|}{\left|H(G^{S},n_{x}^{s},1)\right|},$$

where $\left|H\left(G^{S},n_{i}^{s},1\right)\cap H\left(G^{S},n_{x}^{s},1\right)\right|$ defines the number of the same neighbor nodes of the failed physical sensor node and the candidate backup physical sensor node, and $\left|H(G^{S},n_{x}^{s},1)\right|$ represents the number of neighbor nodes of the failed physical sensor node.

The higher the reliability of the backup node is, the lower the probability of task interruption during the execution of the task on the backup node is. Thus, the recovery capacity of the backup node $n_{i}^{s}$ to the failed node $n_{x}^{s}$ is expressed as (24).

(24) $$RN\left(n_{i}^{s}\right)=NS\left(n_{i}^{s}\right)\cdot RD\left(n_{i}^{s}\right).$$

The specific process of the failure recovery phase is shown in Algorithm 3. First, for multiple affected VSN caused by the same fault physical sensor node, VSN with high penalty shall be restored in priority. Then, for one invalid VSN, virtual sensor nodes are divided into critical and normal virtual sensor nodes according to the VSN topology. Considering the detection range constraints, connectivity constraints of critical virtual sensor nodes and the available resource constraints on physical sensor nodes, a candidate set of backup nodes is constructed. In this set, the physical sensor node with the strongest recovery capability is selected as the backup node. When a physical sensor node fails, fault recovery is performed according to the type of corresponding virtual sensor node failure. If the critical virtual sensor node is embedded in the physical node, it will immediately switch to the reserved backup physical sensor node, otherwise, find feasible nodes in the remaining WSN network for normal virtual sensor node’s fault recovery following the backup node selection principle.
**Algorithm 3** Failure recovery algorithm.**Input:** physical WSN $G^{S}$; VSNR $G_{T}^{V}$; node mapping results set $NODE=\{n_{j}^{s},\cdots ,n_{k}^{s}\}$;Fault physical sensor node $n_{x}^{s}$**Output:** Failure recovery result $RM=\{n_{1}^{s},\cdots n_{m}^{s}\}$**Initialization:** Failure recovery result RM=∅**if**$n_{x}^{s}$ fault **then**      Rank invalid m VSNs in descending order by penalty**end if****for**i=1,⋯,m**do**         
$G^{S}\leftarrow G^{S}/\left\{n_{x}^{s},\;l_{xi}^{s},\;l_{ix}^{s}\right\}$
          **for** $n_{i}^{v}$ in VSNi **do**             **if** $n_{i}^{v}$ is critical virtual sensor node **then**               Remap $n_{j}^{v}$ to $n_{i}^{s}$ through formula (24)               Add $n_{i}^{s}$ to RM         
**else**
            find feasible nodes $n_{m}^{s}$ in the remaining WSN network            Add $n_{m}^{s}$ to RM          **end if**
       **end for****end for****return** the physical node failure recovery successfully

## 5. Analysis of Numerical Results

### 5.1. Parameter Setting

This paper considers the online version of VSN mapping. The VSNRs arrive randomly following a Poisson distribution and each VSNR is processed in order of arrival time and mapped to the underlying physical WSN network. We use matlab2016 for experiments, and the specific parameter settings are shown in Table 1. VSNR arrives following Poisson distribution with an arrival rate of 2 VSNRs. The duration of each VSNR follows the exponential distribution with a mean of 40-time units. As for a VSN, the number of VSN nodes follows the uniform distribution between 3–5, the CPU resources and storage resources requested follow the uniform distribution between 10–20 units, and the virtual link bandwidth resources follow the uniform distribution between 10–25 units. The node fault arrival of the underlying physical sensor network is also subject to Poisson distribution, with an average of two node faults per 100 units of time. The average repair time of the failure is defined as a geometric distribution with a parameter of 10. The substrate WSN network with 50 sensor nodes is generated whereby all the sensor nodes are distributed in a 100×100 (m${}^{2}$) area. The CPU resources and storage resources of physical sensor nodes follow the uniform distribution between 40–80 units, and the physical link bandwidth resources follow the uniform distribution between 40–80 units.

Since the existing VSN mapping method does not consider the physical network node failure, it is inconvenient to directly compare it with the method proposed in this paper. Hence, we expand the classic two-stage mapping algorithm [27] to the GNV algorithm under the node failure environment, which serves as a benchmark comparison algorithm. We change the backup mode on the basis of the reliability-aware embedding algorithm proposed in this paper to compare the performance. The specific algorithm description is shown in Table 2.

### 5.2. Analysis of Experimental Results

**Figure 2:**
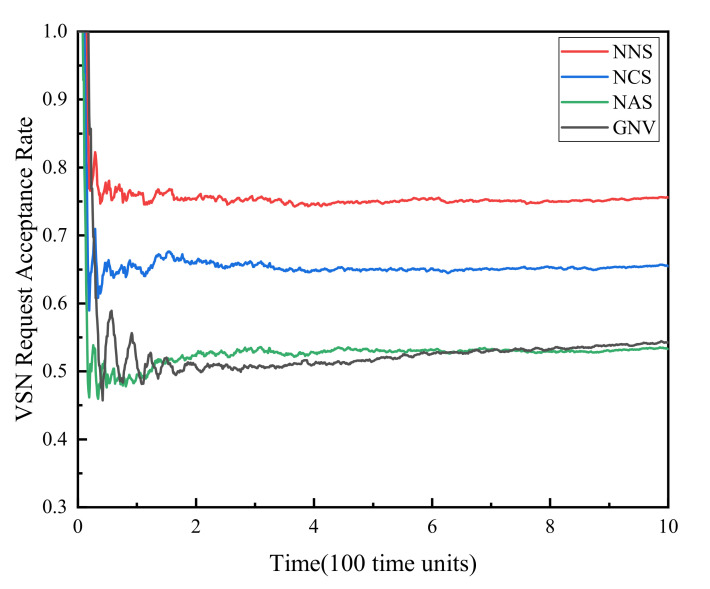
VSNR acceptance rate.

**Figure 3:**
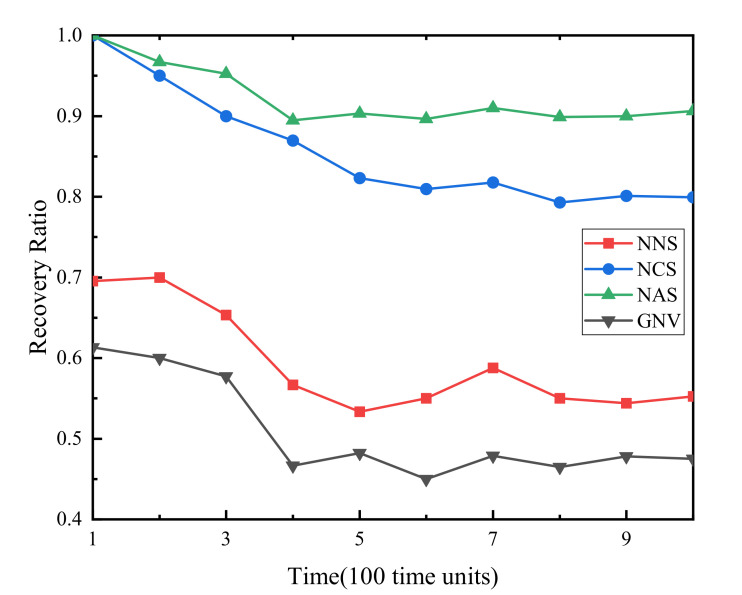
Failure virtual sensor network (VSN) recovery rate.

**Figure 4:**
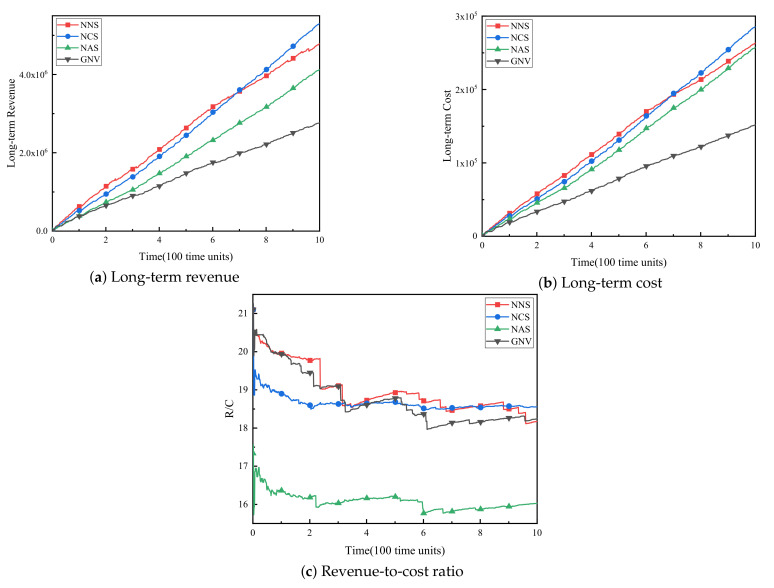
Wireless sensor network infrastructure provider (WSNInP) long-term operating income.

(1) VSNR acceptance rate. Figure 2 shows the comparison of the virtual sensor network request acceptance rate of the four algorithms. It can be seen from the figure that the VSNR acceptance rates of the four algorithms gradually decrease from 1 and tend to be stable at 0.75, 0.65, 0.53, 0.54 over time, respectively. The decline in the acceptance rate is due to the gradual occupation of resources as VSNRs continue to arrive. As the VSN that has completed the sensing task leaves the network, the occupation and release of physical resources reach a dynamic balance, making the acceptance rate reach a steady state. Comparing the four algorithms, NNS has the highest acceptance rate because this algorithm improves the resource allocation method and does not consider resource reservation for failure recovery. The acceptance rate of NCS is second, owing to that, the algorithm considers the reservation of key resources on the basis of improving the resource allocation method, and achieves a compromise between reliability and effectiveness. The NAS algorithm and the GNV algorithm achieved the lowest acceptance rates due to backup resource occupation and unreasonable mapping methods respectively.

(2) Failure VSN recovery rate. Figure 3 demonstrates that the failure recovery rate of the three algorithms proposed in this paper increases by 45%, 35% and 10% respectively compared with the benchmark algorithm. NAS has the highest recovery rate because there is no resource conflict between the backup resources of the full backup strategy. The reason why NASs̀ recovery rate remains at about 0.9 but does not reach 1 is that because of the geographical location of physical WSN nodes, some physical sensor nodes may not be able to find suitable nodes around to back up corresponding virtual sensor nodes. The recovery rate of NCS is stable at around 0.8, which is 0.1 lower than NAS. However, the backup resources consumed by NCS are about 1/3–1/2 of that of NCS, which greatly reduces the waste of physical WSN resources and improves resource utilization. For NNS and GNV algorithms that do not backup resources, with the continuous arrival of VSNR, the idle node resources of WSN gradually decrease, so that the failure recovery rate rapidly decreases to about 0.55, 0.45, respectively.

(3) WSNInP long-term operating income. Figure 4a–c respectively show the long-term revenue, long-term cost and revenue-cost ratio (R/C) of WSNInP for these four algorithms. As shown in Figure 4a,b, the long-term revenues and expenditures of all four algorithms increase over time. Obviously, no matter what kind of backup method is adopted, the long-term revenue of the reliability-aware virtual sensor network embedding algorithm proposed in this paper is much higher than the benchmark algorithm GNV. NCS ultimately has the highest long-term benefits due to its high acceptance rate and high failure recovery rate, while NNS earns less because of the massive fines. As for the NAS algorithm and the GNV algorithm, both of them obtain low benefits because of the low acceptance rate. The long-term cost trends of the four algorithms and the reasons for these trends are the same as the long-term benefits.

The R/C is an important indicator to measure the profitability of WSNInP. The ratio of the NCS is the most stable and relatively high among the four algorithms, indicating that the NCS algorithm is more able to achieve long-term stable high operating income in a fault environment than the other three algorithms.

**Figure 5:**
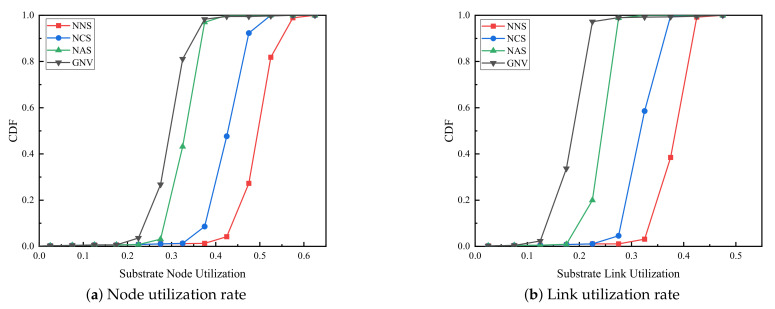
Resource utilization.

(4) Resource utilization. As shown in Figure 5, resource utilization under four algorithms is compared. Figure 5a,b are node resource utilization and link resource utilization respectively. It is obvious that compared with the GNV benchmark algorithm, no matter what kind of backup method is adopted, the node resource utilization rate and link resource utilization rate of the reliability-aware virtual network embedding algorithm proposed in this paper are always higher. With the different backup methods, the resource utilization of full backup, critical node backup and no backup algorithm are improved gradually. The prevalent node utilization ranges are [0.21,0.35] for GNV, [0.27,0.4] for NAS, [0.37,0.4] for NCS and [0.41,0.5] for NNS, which shows the more VSN accepted, the more node resources are utilized. The trend with four algorithms is the same as that with the corresponding node utilization, which shows [0.15,0.25] for GNV, [0.23,0.3] for NAS, [0.27,0.37] for NCS and [0.32,0.45] for NNS respectively.

However, through the analysis of resource utilization rate and WSNInP long-term returns shown in Figure 4, it can be found that although the resource utilization rate of NNS algorithm is higher than that of NCS algorithm, the long-term revenue of the former is gradually lower than that of the latter over time, indicating that in a faulty environment, as the physical sensor node fails, many VSNs adopted NNS algorithm fail during the task execution period and fail to restore due to the physical node failure, which demonstrate that NCS algorithm can guarantee the survivability of VSN better than NNS algorithm. Meanwhile, the NCS algorithm ensures the survivability of VSN with a higher resource utilization rate than the benchmark algorithm, which improves the long-term operating income of WSNInP.

## 6. Conclusions

To make the VSN embedding model satisfy the actual situation, this paper formulates a mixed-integer programming model for the VSN embedding problem with resource constraints, and presents a novel survivability virtual sensor network embedding strategy NCS for WSN virtualization towards physical sensor node failures. Firstly, the VSN is reliably embedded before the physical sensor node fails. Secondly, the critical and normal virtual sensor node is recognized according to the degree of the virtual sensor nodes. Then a fault recovery mechanism based on resource reservation for critical sensor nodes is adopted when a physical sensor fails. Finally, the results of the proposed algorithm have been compared with the benchmark algorithm, the algorithm NCS proposed in this paper shows excellent performance. Specifically, the VSNR acceptance rate and the VSN failure recovery rate increased by 11.27% and 35%, respectively. WSNInP’s long-term revenue has approximately doubled, and its R/C has increased by approximately 30%. The node resource utilization rate and link resource utilization rate increased by 16% and 5% respectively. In addition, in our future work, how to guarantee VSN survivability effectively in multi-node simultaneous failure scenarios will be studied.

## Figures and Tables

**Table 1:** Parameter settings.

Parameters	Value
number of physical sensor nodes	50
simulation area $\left(m^{2}\right)$	100×100
physical node CPU resources $c(n_{i}^{s})$	[40,80]
physical node storage resources $m(n_{i}^{s})$	[40,80]
physical link bandwidth $b(l_{ij}^{s})$	[40,100]
number of VSNR virtual nodes	[3,5]
VSNR virtual node CPU $c(n_{i}^{v})$	[10,20]
VSNR virtual stroage CPU $m(n_{i}^{v})$	[10,20]
VSNR virtual link bandwidth $b(l_{ij}^{v})$	[10,25]
VSNR arrival rate	2
VSNR duration	40
failure happening rate	0.02
mean time to recovery	10
unit price of resource income $p_{price}$	40
unit price of resource cost $p_{cost}$	1.5
node failure rate $\lambda_{i}$	0.01
critical node determination coefficient m	1

**Table 2:** Four embedding algorithms.

Algorithm	VSN Embedding Method	Node Backup Method	Failure Recovery Method
NNS	Node reliability-	No backup	Find a feasible migration node
	aware node embedding		
NCS	Node reliability-		Switch the fault critical virtual node
	aware node embedding	Critical node backup	to backup node or search for a
			feasible node for common virtual node
NAS	Node reliability-	Full backup	Switch the fault virtual
	node selecting with		node to the backup node
GNV	the strongest CPU capability;	No backup	Find a feasible migration node
	selects the shortest path		

## Data Availability

The data presented in this study are available on request from the corresponding author. The data are not publicly available due to further study will be carried out using the same data.
